# Prognostic Significance of Metabolic Parameters by ^18^F-FDG PET/CT in Thymic Epithelial Tumors

**DOI:** 10.3390/cancers13040712

**Published:** 2021-02-09

**Authors:** Joohee Lee, Young Seok Cho, Jhingook Kim, Young Mog Shim, Kyung-Han Lee, Joon Young Choi

**Affiliations:** 1 Department of Nuclear Medicine, CHA Ilsan Medical Center, CHA University, Goyang 10414, Korea; ljhnm04@chamc.co.kr; 2 Department of Nuclear Medicine, Samsung Medical Center, Sungkyunkwan University School of Medicine, Seoul 06351, Korea; ysnm.cho@samsung.com (Y.S.C.); khnm.lee@samsung.com (K.-H.L.); 3 Department of Thoracic Surgery, Samsung Medical Center, Sungkyunkwan University School of Medicine, Seoul 06351, Korea; jhingook.kim@samsung.com (J.K.); youngmog.shim@samsung.com (Y.M.S.)

**Keywords:** thymic epithelial tumor, ^18^F-Fluorodeoxyglucose, PET/CT, standardized uptake value, prognosis

## Abstract

**Simple Summary:**

Thymic epithelial tumors have variable prognoses that depend on histological subtype, and ^18^F-fluorodeoxyglucose positron emission tomography/computed tomography (^18^F-FDG PET/CT) currently plays an important part in oncology images. Thus, we prosecuted a retrospective review of data from 83 patients with thymic epithelial tumors who underwent pretreatment ^18^F-FDG PET/CT and investigated the prognostic significance along with WHO classification, Masaoka stage, and volumetric ^18^F-PET parameters. Masaoka stage, histologic type, treatment modality, maximum standardized uptake values (SUV_max_), average standardized uptake values (SUV_avg_), metabolic tumor volume (MTV), and total lesion glycolysis (TLG) were significant prognostic factors for time-to-progression on univariate survival analysis. On multivariate analysis, SUV_avg_ and Masaoka stage were important independent prognostic factors for progression-free survival in thymic epithelial tumors.

**Abstract:**

Background: Imaging tumor FDG avidity could complement prognostic implication in thymic epithelial tumors. We thus investigated the prognostic value of volume-based ^18^F-fluorodeoxyglucose (^18^F-FDG) positron emission tomography (PET)/CT parameters in thymic epithelial tumors with other clinical prognostic factors. Methods: This is a retrospective study that included 83 patients who were diagnosed with thymic epithelial tumors and underwent pretreatment ^18^F-FDG PET/CT. PET parameters, including maximum and average standardized uptake values (SUV_max_, SUV_avg_), metabolic tumor volume (MTV), and total lesion glycolysis (TLG), were measured with a threshold of SUV 2.5. Univariate and multivariate analysis of PET parameters and clinicopathologic variables for time-to-progression was performed by using a Cox proportional hazard regression model. Results: There were 21 low-risk thymomas (25.3%), 27 high-risk thymomas (32.5%), and 35 thymic carcinomas (42.2%). Recurrence or disease progression occurred in 24 patients (28.9%). On univariate analysis, Masaoka stage (*p* < 0.001); histologic types (*p* = 0.009); treatment modality (*p* = 0.001); and SUV_max_, SUV_avg_, MTV, and TLG (all *p* < 0.001) were significant prognostic factors. SUV_avg_ (*p* < 0.001) and Masaoka stage (*p* = 0.001) were independent prognostic factors on multivariate analysis. Conclusion: SUV_avg_ and Masaoka stage are independent prognostic factors in thymic epithelial tumors.

## 1. Introduction

Thymic epithelial tumors have variable prognoses according to World Health Organization histological subtypes A, AB, B1, B2, B3, and thymic carcinoma. Thymoma types A and AB are generally considered benign tumors; type B1 is a low-grade malignant tumor (10-year survival rate of 90%); type B2 shows a higher degree of malignancy; and type B3 shows the advanced stage and a poor prognosis, similar to that of thymic carcinoma [1].

Differentiation of benign tumors from malignancies is crucial to determining therapeutic options and predicting prognosis. Thymic epithelial tumors are diagnosed by using morphologic examinations, computed tomography (CT), and magnetic resonance imaging (MRI), both of which are good for identifying mediastinal tumors and defining the extent of the tumors [2,3,4]. However, although the conventional image examinations are commonly used for diagnosis and staging with characteristic findings [5,6], these modalities could not fully distinguish histologic subtypes and predict prognosis [7]. In the last decade, several studies have demonstrated the potential benefit of ^18^F-flurorodeoxyglucose (^18^F-FDG) positron emission tomography (PET) or PET/CT for diagnosis, staging, and assessing prognosis in thymic epithelial tumors [6,8,9,10,11,12,13]. These previous reports have concentrated on the visual assessment and semiquantitative value of standardized uptake value (SUV), a widely accepted functional parameter derived from PET, to differentiate subgroups and analyze the prognostic capability of thymic epithelial tumors. However, the evaluations were limited and based only on the parameter of SUV_max_ of ^18^F-FDG PET/CT for pretreatment evaluation and prognostic prediction of thymic epithelial tumors [2,11,12,13]. Currently, three-dimensional volumetric parameters of ^18^F-FDG PET/CT have been proposed as the imaging biomarkers of malignancy patients [14,15,16,17]. It is expected to assist in measuring the volumetric tumor burden of the metabolic activity, delineated as metabolic tumor volume (MTV) or total lesion glycolysis (TLG). In recent studies, these volumetric parameters have been suggested to be independent factors of poor prognosis in some malignancies [18,19,20,21,22,23]. However, the volumetric parameters of PET have rarely been studied in patients with thymic epithelial tumors. Although a previous study showed an association of volume-dependent ^18^F-FDG PET/CT parameters with proposed prognostic factors, including WHO classification and Masaoka stage [24], there were no results regarding the prognostic value of ^18^F-FDG PET/CT due to the short follow-up duration. The study could not evaluate the clinical follow-up data of the prognosis on SUV_max_ and TLG since this would require long term follow-up periods.

Therefore, in this retrospective study, we investigated the prognostic value of volume-based metabolic parameters by ^18^F-FDG PET/CT in thymic epithelial tumor patients for the stratification of the disease outcome early in the course of treatment.

## 2. Materials and Methods

### 2.1. Patient Population

A total of 83 patients with pathologically confirmed thymic epithelial tumors and who underwent ^18^F-FDG PET/CT pretreatment at the Samsung Medical Center were enrolled in this retrospective cohort study. Our Institutional Review Board approved this retrospective study, and the requirement to obtain informed consent was waived. ^18^F-FDG PET/CT images, medical records, and pathologic data were retrospectively reviewed.

### 2.2. Histology

Histological and immunohistochemical interpretations were determined by experienced pathologists [25]. All cases were classified according to WHO classification as low-risk thymoma (A, AB, B1), high-risk thymoma (B2, B3), and thymic carcinoma (C) [25]. Pathology results were divided using the Masaoka stages: I, macroscopically completely encapsulated and microscopically no capsular invasion; II, microscopic invasion into the capsule and macroscopic capsular invasion into surrounding fatty tissue or mediastinal pleura; III, macroscopic invasion into the neighboring organ; IVa, pleural or pericardial dissemination; and stage IVb, lymphogenous or hematogenous metastasis [20].

### 2.3. PET/CT Imaging

All patients fasted for at least 6 h before PET/CT scans, and the serum glucose level at the time of injection of ^18^F-FDG was <200 mg/dL. PET/CT was without contrast on the GE Healthcare (Milwaukee, WI) Discovery LS scanners. Images were acquired from skull base to mid-thigh 60 min after injecting 5.5 MBq/kg FDG for 4 min per frame in 2D mode. Whole-body spiral CT was performed with an 8-slice helical CT (140 KeV, 40–120 mAs adjusted to body weight; section width = 5 mm). Attenuation-corrected PET images (voxel size = 3.9 × 3.9 × 3.3 mm) were reconstructed using CT data, and a 2D ordered subsets expectation maximization algorithm (28 subsets, 2 iterations), and displayed on a 128 × 128 matrix with a voxel size of 4.3 × 4.3 × 3.9 mm^3^.

### 2.4. Measurements of Metabolic PET Parameters

**Figure 1:**
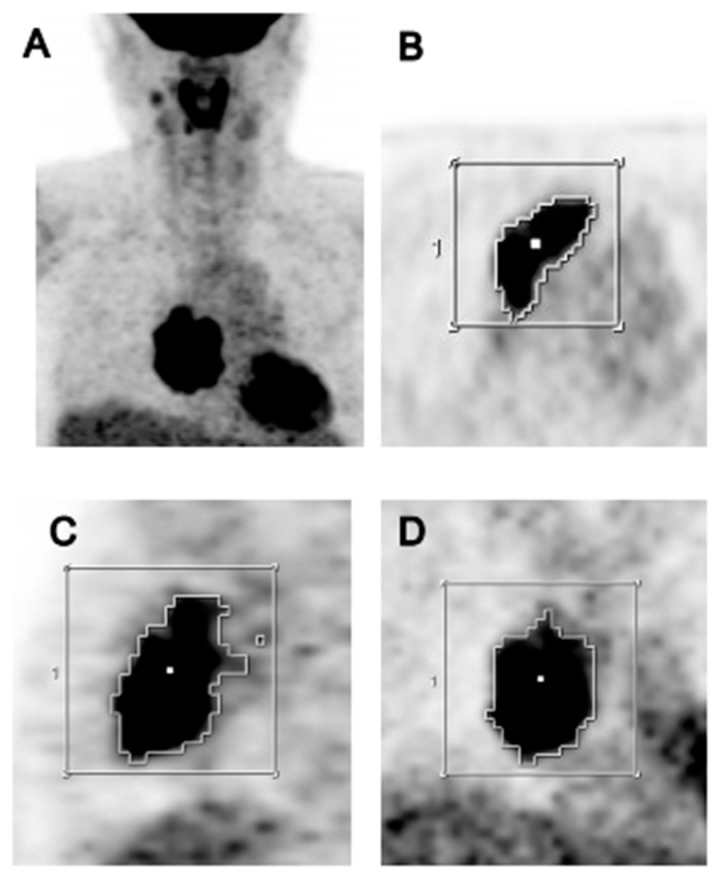
^18^F-fluorodeoxyglucose positron emission tomography/computed tomography (^18^F-FDG PET/CT) images of a 54-year-old female patient with thymoma type B1. (**A**) The high FDG uptake by the primary mediastinal tumor is clearly visible in the maximum intensity projection image. A volume of interest (VOI) was semi-automatically placed over the tumor using an isocontour threshold of SUV 2.5. The segmented VOI is shown on the transverse (**B**), sagittal (**C**), and coronal (**D**) images.

Semiquantitative and volumetric measurements were conducted using volume viewer software on a dedicated workstation (GE Advantage Workstation 4.4), which provided a semi-automatic method to delineate the volume of interest (VOI) using an isocontour threshold method based on the SUV. MTV was defined as the total tumor volume segmented by the threshold SUV (Figure 1) [18]. A standard method for the determination of the optimal threshold has not been established, although many methods have been suggested [26]. In this study, a threshold SUV of 2.5 was used for the tumor segmentation because this value showed the highest statistical significance in predicting progression. Maximum SUV (SUV_max_), average SUV (SUV_avg_), MTV, and TLG of tumor VOIs derived using these thresholds were measured.

### 2.5. Statistical Analysis

All quantitative data are expressed as mean ± standard deviation (SD). Time-to-progression (TTP) was defined as the elapsed time between the date of initial diagnosis and the date of detection of recurrence or progression or the date of death attributable to a thymic epithelial tumor. Patients with no evidence of progressive disease were censored at the date of the final follow-up study. The relation between clinicopathological tumor characteristics, PET-derived parameters, and progression-free survival (PFS) was assessed by univariate and multivariate analyses using a Cox proportional hazard regression model. Hazard ratios (HRs) and associated 95% confidence intervals (CIs) were calculated. In addition, binary logistic regression analysis was used for establishing survival curves for several significant variables. Predicted probability yielded an area under the receiver operating characteristics (ROC) curve as an index of prognostic performance of logistic models. The significance of differences in variables was tested using a *t*-test for 2 groups and analysis of variance (ANOVA) for 3 groups. Data management and statistical analyses were compiled using MedCalc (MedCalc Software, Mariakerke, Belgium) and SPSS Statistics 19 (IBM Corporation, Somers, NY, USA). *p* < 0.05 was regarded as statistically significant.

## 3. Results

### 3.1. Clinical Characteristics and Metabolic Parameters

Patients’ characteristics are summarized in detail in Table 1. Among a total of 83 patients, there were 46 males (55.4%) and 37 females (44.6%) with a mean age of 51.7 ± 12.9 years (range, 15–77 years). There were 21 low-risk thymomas (25.3%), 27 high-risk thymomas (32.5%), and 35 thymic carcinomas (42.2%) determined by WHO classification methods. The Masaoka stage was I in 14 (16.9%), II in 25 (30.1%), III in 9 (10.8%), IVa in 12 (14.5%), and IVb in 23 (27.7%). Sixty (72.3%) of the 83 patients underwent surgical resection; the remaining 23 (27.7%) had non-surgical treatment, including chemotherapy (*n* = 13), radiation therapy (*n* = 3), or both (*n* = 7). Of the 60 patients undergoing surgical resection, 31 (51.7%) underwent postoperative adjuvant therapy. This included radiation therapy (*n* = 19), chemotherapy (*n* = 6), and chemoradiotherapy (*n* = 6). In all patients, mean SUV_max_, SUV_avg_, MTV, and TLG were 8.0 ± 5.7 (range, 2.5–41.0), 4.2 ± 1.6 (range, 2.5–11.5), 61.9 ± 63.0 (range, 0.1–359.0), and 312.1 ± 379.1 (range, 0.2–1866.8), respectively.

The mean clinical follow-up period was 28.6 ± 22.2 months (range, 0.0–79.0 months). By the last follow-up date, twenty-four patients (28.9%) developed disease progression. Of these, two were low-risk thymomas (8.3%), six were high-risk thymomas (25.0%), and sixteen were thymic carcinomas (66.7%). Eight patients had died of the thymic epithelial tumors.

### 3.2. PET Metabolic Parameter Evaluation and Grade Based on WHO Classification and Masaoka Stage of the Thymic Tumors

The relationships between PET parameters and the WHO classification and Masaoka stage are illustrated in Table 2. Mean (SD) SUV_max_ was 4.8 ± 2.0 in low-risk thymomas, 5.5 ± 2.2 in high-risk thymomas, and 11.8 ± 6.7 in thymic carcinomas. These differences were statistically significant (*p* < 0.001). Mean (SD) SUV_avg_ was 3.2 ± 0.8 in low-risk thymomas, 3.5 ± 0.9 in high-risk thymomas, and 5.3 ± 1.8 in thymic carcinomas. These differences were also statistically significant (*p* < 0.001). Mean (SD) MTV and TLG were 26.0 ± 29.6 and 99.3 ± 133.0, respectively, in low-risk thymomas, 47.2 ± 42.9 and 218.6 ± 210.9 in high-risk thymomas, and 94.7 ± 74.7 and 512.0 ± 475.6 in thymic carcinomas (both *p* < 0.001). On evaluation by Masaoka stage, mean (SD) SUV_max_ was 6.6 ± 4.8 in I, 4.9 ± 1.8 in II, 8.9 ± 5.7 in III, and 10.5 ± 6.7 in IV; the differences were statistically significant (*p* = 0.001). Mean (SD) SUV_avg_ was 3.3 ± 1.0 in I, 3.7 ± 1.1 in II, 3.8 ± 1.2 in III, and 4.9 ± 1.9 in IV; these differences were statistically significant (*p* = 0.003). Mean (SD) MTV and TLG were 36.5 ± 46.0 and 150.1 ± 221.8, respectively, in I, 39.8 ± 36.6 and 166.5 ± 162.0 in II, 39.2 ± 51.1 and 191.2 ± 293.6 in III, and 93.6 ± 73.6 and 512.0 ± 466.5 in IV. The differences in both parameters were statistically significant (*p* < 0.001).

### 3.3. Prognostic Analyses

**Figure 2:**
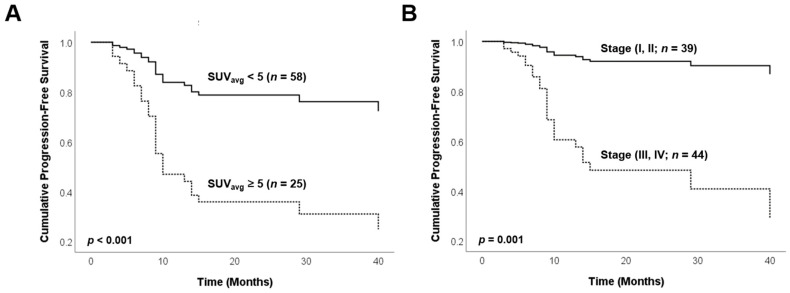
Progression-free survival curves of study subjects sub stratified according to standardized uptake values Average (SUV_avg_) (**A**) and Masaoka stage (**B**): SUV_avg_ classified into high/low with 5.0 as a cut-off and Masaoka stage classified into I, II and III, IV.

Univariate survival analysis showed that the Masaoka stage, histologic type, treatment modality, SUV_max_, SUV_avg_, MTV, and TLG were significant prognostic factors for time-to-progression (Table 3). Age and gender were not statistically significant factors for prognosis in the univariate analysis. Multivariate survival analysis adjusted for age, histologic type, treatment modality, and PET parameters showed that SUV_avg_ (*p* < 0.001, hazard ratio (HR) = 1.459) and Masaoka stage (*p* = 0.001, HR = 9.060) were independent factors associated with disease progression (Table 4). Kaplan–Meier time-to-progression analysis was performed in two subgroups, patients with Masaoka stages I and II tumors (subgroup 1) and patients with Masaoka stages III and IV tumors (subgroup 2) (Table 4). Survival curves showed significantly worse prognoses with Masaoka stages III and IV and higher SUV_avg_ than those with stages I and II and lower SUV_avg_ (Figure 2).

## 4. Discussion

Tumor FDG avidity assessed by PET/CT imaging provides information regarding the biological behavior of thymic tumors. This study investigated the relative prognostic values of PET parameters with metabolic values in thymic epithelial tumors, and the results showed a statistically significant relationship between PET parameters and patients’ outcomes. Additionally, Masaoka stage and SUV_avg_ were independent prognostic factors associated with tumor progression in this study.

Several studies have reported the evaluation of ^18^F-FDG PET/CT images in thymic epithelial tumors. Liu et al. first reported ^18^F-FDG avidity in thymomas and the usefulness for assessing invasiveness [27]. Sung et al. suggested that SUV_max_ is significantly higher in thymic carcinoma than thymomas [11]. They also reported that a higher proportion of thymic carcinoma patients show a more homogeneous ^18^F-FDG avidity than thymoma patients [11]. In accordance with a previous study, Kim et al. showed that image findings of ^18^F-FDG PET or ^18^F-FDG PET/CT differed by histologic classifications, including SUV_peak_ visual uptake grading, uptake pattern, and contour [12]. Kumar et al. and Endo et al. showed ^18^F-FDG PET/CT can help characterize various thymic lesions by using SUV_max_ and its tumor to mediastinum (T/M) ratio [13]. However, there are scarce data concerning the relationships between metabolic PET parameters and thymic epithelial tumors. Park SY et al. informed that a remarkable relationship was showed between SUV_max_ and WHO classification and Masaoka stage, but the metabolic parameters were not correlated [24]. These researchers analyzed the relationships of SUV_max_, TLG, and MTV, but not SUV_avg_, and a small number of cases of thymic carcinoma.

The main goal of this study was to determine the relative prognostic values of metabolic parameters, including SUV_max_, SUV_avg_, MTV, and TLG, in a thymic epithelial tumor sample with a reasonable proportion of thymic carcinomas. The results showed that SUV_avg_ and Masaoka stage were independent prognostic factors. Univariate analysis demonstrated that higher tumor PET parameters were strong predictors of progression-free survival, along with Masaoka stage, WHO classification, and treatment modalities. Multivariate regression analysis, including these variables adjusted for age, revealed that high SUV_avg_ (>5.0) and Masaoka stage were independent predictors of poor survival. Although WHO classification is used for the histologic classification of thymomas, the prognostic significance has been controversial. Masaoka staging system, in this sense, has been used most widely to determine further treatment and to predict prognosis, and it is in line with the conclusions reached in our study. The identification of high SUV_avg_ (>5.0) as a significant independent predictor of poor survival in patients with thymic epithelial tumors is a key finding of this study. A previous study showed that homogenous ^18^F-FDG uptake was observed in the order low-risk thymoma, high-risk thymoma, thymic carcinoma [11]. The characteristic of heterogeneous ^18^F-FDG uptake exists, especially in lower histologic grade thymic epithelial tumors, and SUV_avg_ might better reflect tumor characteristics than SUV_max_. Our result indicates that tumor SUV_avg_ can be used to stratify prognosis in thymic epithelial tumors.

There were 35 confirmed cases of thymic carcinomas with 7 deaths, which covered almost all the deaths of the enrolled population (7/8, 87.5%). Only one death was counted in high-risk thymoma. When arranged in increasing order of SUV_avg_ in the thymic cancer group, all of the events were observed over SUV_avg_ 5.6 from 5.0 to 11.5. However, only three of seven death cases underwent palliative treatment though the data showed the largest percentage of palliative treatment consisted of the thymic cancer group (Low-risk thymoma 4.8% (1/21); high-risk thymoma 18.5 (5/27); thymic carcinoma 48.6% (17/35)). As it is hard to show statistical significance because of the small number of cases, a more tailored therapy strategy might be necessary to meet a better prognosis in the case of higher SUV_avg_, even operable cases. It is recommended that further investigations should be conducted with a large number of cases to verify the hypothesis and to get a valid cut-off value of SUV_avg_.

SUV_max_, a semiquantitative index for tumor ^18^F-FDG uptake, was shown to be a valuable parameter for the prediction of histopathologic type and to be a potential prognostic factor in thymic epithelial tumors. In this study, SUV_max_ had a significant prognostic value for progression-free survival in univariate analysis but was not an independent prognostic factor in multivariate analysis. Obtained for the 1-pixel region of interest (ROI), SUV_max_ depended strongly on noise and, in high-noise situations, behaved in an unpredictable manner. In addition, even without noise, a single pixel may not be representative of the overall tumor uptake in a non-homogeneous tumor [11,28,29].

TLG and MTV are three-dimensional volumetric measurements incorporated with metabolic activity. While SUV_max_ did not represent total tumor mass, volume-based measurements reflected the metabolically active tumor cells. Therefore, MTV and TLG are theoretically more relevant methods than the single pixel value. Therefore, these volume-based parameters provide valuable information by representing tumor burden and aggressiveness and are important prognostic factors in various tumors. In patients with thoracic tumors, such as esophageal cancer, lung cancer, and malignant mesothelioma, a number of studies that evaluated and compared the prognostic value of these parameters have been conducted [16,18,19,30,31,32]. To our knowledge, however, there is no study that statistically evaluated the prognostic value of these parameters in patients with thymic epithelial tumors. In this study, MTV and TLG were univariate variables associated with poor survival, but there were no statistically significant differences in multivariate analysis. This may be related to heterogeneous tumor uptake in large-sized thymic epithelial tumors as the result of necrosis, fibrosis, or hemorrhage. Therefore, MTV and TVG may overestimate actual metabolic activity [3,33,34,35].

This study has several limitations. First, the study was a retrospective review with various treatment protocols. The various options of treatment may have a confounding effect on prognostication. In addition, since the relatively small number of patients were included in this evaluation, it might be an obstacle to the generalization and application of these results. Therefore, large-sized prospective validation studies with a homogeneous population of thymic epithelial tumor patients are needed. Second, the authors did measure primary tumor burden, except metastatic lesions. Third, the incidence of thymic carcinoma was higher than those previously known for thymomas. This is because surgical management or simple clinical follow-up without PET/CT is given first if the lesion is a small mediastinal mass suspected of being a low-risk thymoma or another benign tumor. Another possible cause is that our institution corresponds to a tertiary hospital, the medical institution with the highest level in our country, which might contribute to the high disease severity of the patients. Last, because of the slow-progressing behavior of thymic epithelial tumors, overall survival was not used as the primary outcome for survival. Further studies with a large number of cases and longer follow-up periods are warranted to clearly elucidate the prognostic significance of the PET parameters, including overall survival.

## 5. Conclusions

In conclusion, the present study demonstrated that SUV_avg_ and Masaoka stage are important independent prognostic factors for progression-free survival in thymic epithelial tumors. These results suggest that SUV_avg_ might be a potentially valuable parameter for stratification and predicting clinical prognosis. Additional large-scale prospective studies are needed to validate the result of this promising functional biomarker derived from ^18^F-FDG PET/CT in thymic epithelial tumors.

## Figures and Tables

**Table 1:** Patient demographics and clinical characteristics (*n* = 83).

Characteristics	No. (%)
Age, mean (SD), y	51.7 (12.9)
Sex	
Male	46 (55.4)
Female	37 (44.6)
Histologic type (WHO classification)	
Low-risk thymoma (A, AB, B1)	21 (25.3)
High-risk thymoma (B2, B3)	27 (32.5)
Thymic carcinoma (C)	35 (42.2)
Masaoka stage	
I	14 (16.9)
II	25 (30.1)
III	9 (10.8)
IVa/IVb	12 (14.5)/23 (27.7)
Treatment	
Surgery only	29 (34.9)
Surgery and Adjuvant therapy	31 (37.3)
Radiation therapy	19 (61.3)
Chemotherapy	6 (19.4)
Chemoradiotherapy	6 (19.4)
Non-surgical treatment	23 (27.7)
Radiation therapy	3 (13.0)
Chemotherapy	13 (56.5)
Chemoradiotherapy	7 (30.4)

WHO: World Health Organization.

**Table 2:** Positron emission tomography (PET) parameters by Masaoka stage and histologic type (WHO classification).

**PET Parameters**	**Histologic Type (WHO Classification)**				***p*-Value**
	**Low-risk thymoma** **(A, AB, B1)**	**High-risk thymoma** **(B2, B3)**	**Thymic carcinoma (C)**		
SUV_max_	4.8 ± 2.0	5.5 ± 2.2	11.8 ± 6.7		<0.001
SUV_avg_	3.2 ± 0.8	3.5 ± 0.9	5.3 ± 1.8		<0.001
MTV	26.0 ± 29.6	47.2 ± 42.9	94.7 ± 74.7		<0.001
TLG	99.3 ± 133.0	218.6 ± 210.9	512.0 ± 475.6		<0.001
	**Masaoka stage**				
	**I**	**II**	**III**	**IV**	
SUV_max_	6.6 ± 4.8	4.9 ± 1.8	8.9 ± 5.7	10.5 ± 6.7	0.001
SUV_avg_	3.3 ± 1.0	3.7 ± 1.1	3.8 ± 1.2	4.9 ± 1.9	0.003
MTV	36.5 ± 46.0	39.8 ± 36.6	39.2 ± 51.1	93.6 ± 73.6	<0.001
TLG	150.1 ± 221.8	166.5 ± 162.0	191.2 ± 293.6	512.0 ± 466.5	<0.001

SUV_max_: maximum standardized uptake value, SUV_avg_*:* average standardized uptake value, MTV: metabolic tumor volume, TLG: total lesion glycolysis.

**Table 3:** Univariate analysis for time-to-progression using Cox proportional-hazard model.

Variable	HR	95% CI	*p*-Value
Age (1-year increase)	-		0.060
Sex	-		0.716
Masaoka stage (I, II vs. III, IV)			<0.001
Histologic type (thymoma vs. thymic carcinoma)			0.003
Treatment (Surgery and/or adjuvant Tx. vs. Non-surgical)			0.001
SUV_max_ (1-unit increase)	1.111	1.061–1.164	<0.001
SUV_avg_ (1-unit increase)	1.403	1.195–1.647	<0.001
MTV (10-cm^3^ increase)	1.007	1.003–1.012	<0.001
TLG (100-unit increase)	1.002	1.001–1.002	<0.001

HR: Hazard Ratio, SUV_max_: maximum standardized uptake value, SUV_avg_: average standardized uptake value, MTV: metabolic tumor volume, TLG: total lesion glycolysis.

**Table 4:** Multivariate analysis for time-to-progression using a Cox proportional hazard model.

Variable	HR	95% CI	*p*-Value
SUV_avg_ (1-unit increase)	1.459	1.193–1.784	<0.001
Masaoka stage (I, II vs. III, IV)	9.060	2.610–31.447	0.001

HR: hazard ratio, CI: confidence interval, TLG: total lesion glycolysis.

## Data Availability

Restrictions apply to the availability of these data. Data were obtained from the Samsung Medical Center and are available from the corresponding author with the permission of the Samsung Medical Center.
